# Identification of a rare 17p13.3 duplication including the *BHLHA9* and *YWHAE* genes in a family with developmental delay and behavioural problems

**DOI:** 10.1186/1471-2350-13-93

**Published:** 2012-10-04

**Authors:** Valeria Capra, Marisol Mirabelli-Badenier, Michela Stagnaro, Andrea Rossi, Elisa Tassano, Stefania Gimelli, Giorgio Gimelli

**Affiliations:** 1 U.O. Neurochirurgia, Istituto G.Gaslini, Genova, Italy; 2 U.O. Neuropsichiatria, Istituto G.Gaslini, Genova, Italy; 3 Dipartimento di Neuroradiologia, Istituto G. Gaslini, Genova, Italy; 4 Service of Genetic Medicine, University Hospitals of Geneva, Geneva, Switzerland; 5 Laboratorio di Citogenetica, Istituto G. Gaslini, Genova, Italy

**Keywords:** Familial 17p13.3 duplication syndrome, *PAFAH1B1* and *YWHAE* genes, Array-CGH

## Abstract

**Background:**

Deletions and duplications of the *PAFAH1B1* and *YWHAE* genes in 17p13.3 are associated with different clinical phenotypes. In particular, deletion of *PAFAH1B1* causes isolated lissencephaly while deletions involving both *PAFAH1B1* and *YWHAE* cause Miller-Dieker syndrome. Isolated duplications of *PAFAH1B1* have been associated with mild developmental delay and hypotonia, while isolated duplications of *YWHAE* have been associated with autism. In particular, different dysmorphic features associated with *PAFAH1B1* or *YWHAE* duplication have suggested the need to classify the patient clinical features in two groups according to which gene is involved in the chromosomal duplication.

**Methods:**

We analyze the proband and his family by classical cytogenetic and array-CGH analyses. The putative rearrangement was confirmed by fluorescence in situ hybridization.

**Results:**

We have identified a family segregating a 17p13.3 duplication extending 329.5 kilobases by FISH and array-CGH involving the *YWHAE* gene, but not *PAFAH1B1,* affected by a mild dysmorphic phenotype with associated autism and mental retardation. We propose that *BHLHA9*, *YWHAE*, and *CRK* genes contribute to the phenotype of our patient. The small chromosomal duplication was inherited from his mother who was affected by a bipolar and borderline disorder and was alcohol addicted.

**Conclusions:**

We report an additional familial case of small 17p13.3 chromosomal duplication including only *BHLHA9*, *YWHAE,* and *CRK* genes. Our observation and further cases with similar microduplications are expected to be diagnosed, and will help better characterise the clinical spectrum of phenotypes associated with 17p13.3 microduplications.

## Background

The short arm of chromosome 17 is particularly prone to submicroscopic rearrangements due to the presence of high density low copy repeats (LCRs). The proximal region of the short arm harbors a number of syndromes such as CMT1A (Charcot–Marie–Tooth syndrome type 1A), HNPP (hereditary neuropathy with liability to pressure palsies), Smith–Magenis syndrome, and Potocki–Lupski syndrome. It is known that heterozygous 17p13.3 deletions, including *PAFAH1B1* (MIM 601545) and *YWHAE* (MIM 605066) genes, cause two clinically distinct disorders: LSI (isolated lissencephaly) or MDS (Miller-Dieker syndrome), depending on the size of the deletion [1]. Recently, new genomic disorders have been identified in the MDS locus. To date, twenty-two microdeletions [2-6] and sixteen microduplications [5,7-9] overlapping the MDS critical region have been described in unrelated individuals. All these submicroscopic rearrangements are variable in size and have distinct breakpoints. Bruno et al. [5] proposed to divide 17p13.3 microduplications in two different classes: class I microduplications involving *YWHAE* but not *PAFAH1B1* showing a phenotype characterized by learning difficulties and/or autism with or without other congenital abnormalities; class II microduplications always harboring *PAFAH1B1* that may also include the genomic region encompassing the *CRK* and *YWHAE* genes, which are associated with developmental delay, psychomotor delay, and associated hypotonia.

Here we report on a 7-year-old boy and his mother presenting a novel class I 17p13.3 microduplication measuring about 329.5 Kb in length and involving only seven genes, including *YWHAE, CRK,* and *BHLHA9*.

## Methods

### Case presentation

**Figure 1:**
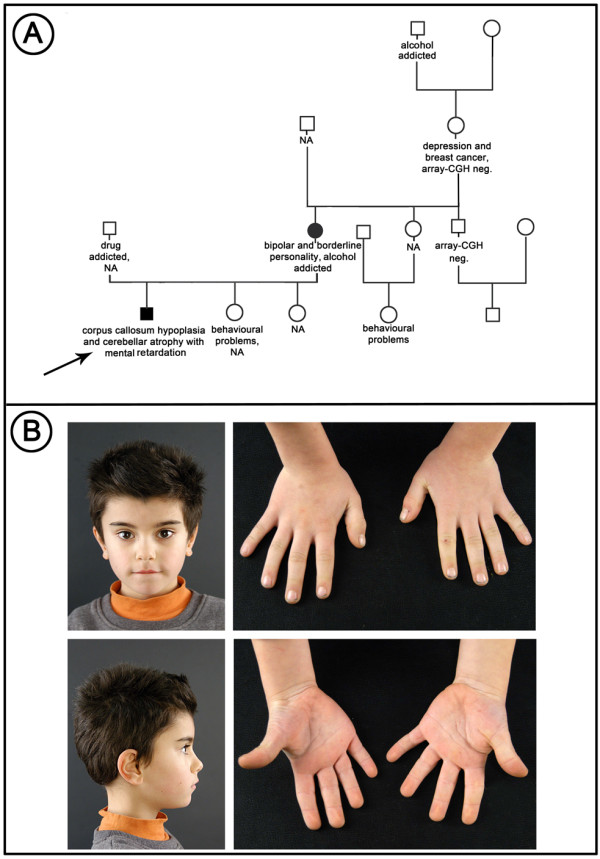
** A) Family tree that shows the individuals who were available for array-CGH.** Some components of the family were reported as being affected by behavioural deficits. Arrow indicates the affected child. Full symbols indicate the individuals carrying the 17p13.3 duplication. NA indicates the individuals not available for array-CGH. **B**) Photographs of face and hands of the child carrying the 17p13.3 duplication. Mild facial anomalies such as thickened eyebrows, upslanting eyes, squared nasal tip, large and low-set ears. Short and stubby hands with low-set thumbs. The step mother gave consent to publish the photoghaphs of the child.

The proband, a 7-year-old boy with a Caucasian ancestry, is the third child of non-consanguineous parents. The mother presents antisocial behaviour and bipolar disorder, she was HIV positive and alcohol addicted. The father was drug addicted, but was not available for array-CGH analysis. One sister was reported as healthy while a second one was reported to manifest behavioural problems and aggressiveness. Both sisters were not available for testing because they were living apart from their brother. The proband is currently legally in care of his maternal grandparents, but lives with his maternal uncle. His maternal grandmother was affected by depression and has been recently diagnosed as having bilateral breast cancer. His maternal grandmother and maternal uncle were available for array-CGH analysis (Figure 1A).

**Figure 2:**
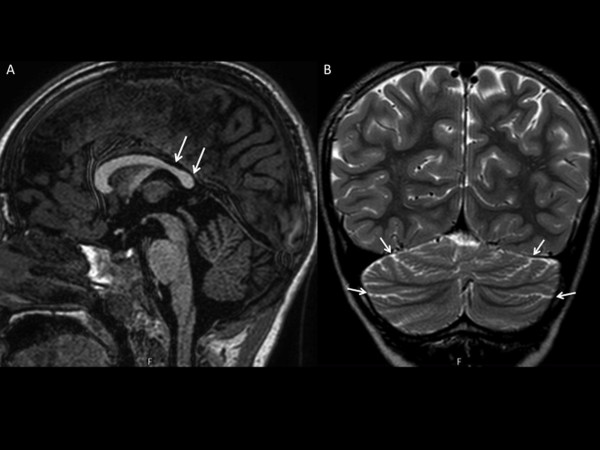
** MR imaging. A**) sagittal turbo-field-echo T1-weighted image shows hypoplastic callosal splenium (arrows). **B**) coronal fast spin-echo T2-weighted image shows slightly prominent cerebellar fissures (arrows), consistent with a mild form of cerebellar hypoplasia.

The child was born at 37 weeks of gestation by caesarean section after a pregnancy complicated by gestosis. His birthweight was 3350 g, length and head circumference were not reported. APGAR score was 8 and 9 at first and fifth minute. At eight months of age, because of motor delay and mild lower limb hypertonia, transfontanellar ultrasound was performed and it appeared normal. The patient underwent physiotherapy treatment for one year with partial improvement. He was able to walk at 18 months of age and uttered his first words at 2 years of age. Moreover, poor social relationships and motor stereotypes were noticed. He presented appropriate growth. At five years five months of age, his neurological examination was characterized by clumsiness without obvious focal signs. A moderate mental retardation was noticed: the General Developmental Quotient assessed according to the Griffiths Mental Development Scale was 46. Behavioural and neuropsychological evaluations revealed an autism defined as a pervasive developmental disorder not otherwise specified (PDD-NOS) with social interaction and communication impairment, motor stereotypes, persistent behaviours, and attention deficit. At seven years five months of age, weight was 22.8 kg (50^th^ centile), height 123 cm (75^th^ centile), and head circumference 52.5 cm (25^th^-50^th^ centile). At physical examination, the child presented thick linearized eyebrows, myopia with upslanting eyes, squared upturned tip of the nose, pointed chin, large and low-set ears. His hands were short and stubby with low-set thumbs (Figure 1B). Both feet presented laterally set fifth toes. Laboratory investigations for metabolic disorders (ammonium, lactic acid, creatine kinase, aminoacids in serum and urine, organic acids and creatine/guanidinoacetate in urine, isoelectric focusing of serum transferrin) were normal. Ophthalmologic evaluation, electroencephalogram and auditory evoked potentials were also normal. Brain MRI performed at five years five months of age revealed reduction of the volume and thickness of the isthmus and of the splenium of the corpus callosum with a dysmorphic aspect of the rostrum. No lesions of the supratentorial tissues were identified. Both cerebellar hemispheric and vermian folia, in the subtentorial regions, were wilted without signal alteration (Figure 2). A control MRI after one year confirmed the presence of non-progressive neuroradiologic features characterized by posterior corpus callosum hypoplasia and mild cerebellar hypoplasia. Molecular analysis for Fragile-X syndrome was negative. The mother was not available for clinical evaluation or MRI screening but consented to cytogenetic analysis.

### Cytogenetic, Fluorescence in situ hybridization and array-CGH analyses

Cytogenetic analysis was performed using GTG-banding techniques on metaphase chromosomes obtained by standard procedures from peripheral blood lymphocytes. FISH analyses, according to standard protocols, were carried out using BlueFISH BAC probes ( http://www.cambridgebluegnome.com/bluefish) RP11-294J5 and RP11-100F18 mapping to 17p13.3 at the positions chr17:1, 199,461-1,352,559 and chr17:1,314,902-1,514,082 (build 37.1, Feb 2009), respectively. Array-CGH was additionally performed on the proband and other family members using the Agilent Human Genome CGH Microarray Kit G3 400K (Agilent Technologies, Santa Clara, CA, USA) platform, according to the manufacturer’s instructions. This high resolution 60-mer oligonucleotide-based microarray spans coding and non coding genomic sequences with a median spacing of 5.3 kb. Data analysis was performed using the Agilent Genomic Workbench Lite Edition Software 6.5.0.18(2) with the following settings for CGH aberration calling: ADM-2 algorithm (threshold 5) with a moving average of 500 KB and visual inspection of the log2 ratios. DNA sequence information was according to the UCSC Genome Browser ( http://genome.ucsc.edu/; GRCh37/hg19, February 2009).

## Results

**Figure 3:**
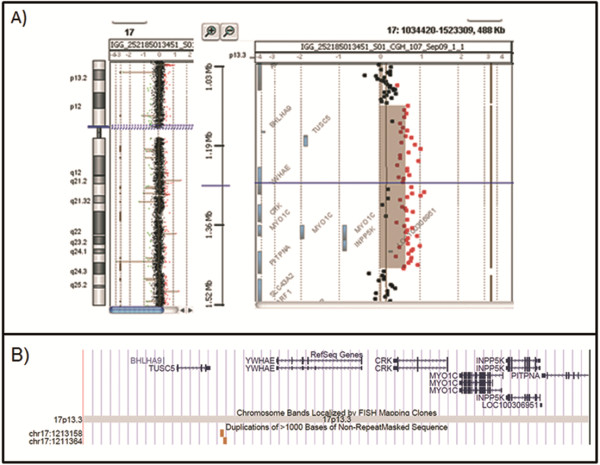
** A) Result of array-CGH analysis of chromosome 17 with Agilent Human Genome CGH microarray Kit G3 400K.** The 17p13.3 duplicated region extends between probes A_18_P12560163 (1,122,235Mb) and A_16_P20564512 (1,451,751Mb). **B**) Gene contents of the duplicated region.

The patient and his mother had a normal G-band karyotype. The array CGH analysis revealed a 17p13.3 duplication of ~329.5 Kb between oligomers at 1,122,235Mb (A_18_P12560163, first duplicated) and 1,451,751Mb (A_16_P20564512, last duplicated), flanked by oligomers at 1,117,136Mb (A_16_P20563792, first present) and 1,455,928Mb (A_16_P03201459, last present), in the patient and his mother, while his maternal grandmother and his maternal uncle had normal array-CGH analysis (Figure 3A). FISH studies with BACs RP11-294J5 and RP11-100F18 excluded a more complex rearrangement and the insertion of the duplicated segment in another chromosome. The duplicated region contains only seven genes: *BHLHA9* (basic helix-loop-helix family, member a9), *TUSC5* (tumor suppressor candidate 5), *YWHAE* (tyrosine 3-monooxygenase/tryptophan 5-monooxygenase activation protein, epsilon polipeptide), *CRK* (v-crk avian sarcoma virus CT10 oncogene homolog), *MYO1C* (MYOSIN 2), *INPP5K* (inositol polyphosphate 5-phosphatase K), *PITPNA* (phosphatidylinositol transfer protein alpha isoform) (intron 9) (Figure 3B).

## Discussion

The use of array-CGH analyses for investigation of children with mental retardation has led to the identification of a growing number of new microdeletion and microduplication syndromes, some of which have been clinically well characterised while some other await further delineation.

The proximal short arm of chromosome 17 is particularly prone to cryptic rearrangements for the presence of a high density of low copy repeats. The Miller-Dieker syndrome (MDS) is localized in the more distal region 17p13.3 containing the *PAFAH1B1* (encoding LIS1) and *YWHAE* genes. Recently, novel co-locating microdeletions and microduplications in chromosome 17p13.3 were identified by array-CGH describing new genomic disorders in the MDS locus. Most of these rearrangements are non-recurring and vary in size, from one hundred kilobases to about three megabases. The clinical characterization of both microdeletions and microduplications has been dissected according to the extension and the genes involved in such rearrangements. The main characteristics of 17p13.3 microdeletion are significant postnatal growth retardation, mild to moderate mental retardation, and facial anomalies.

**Figure 4:**
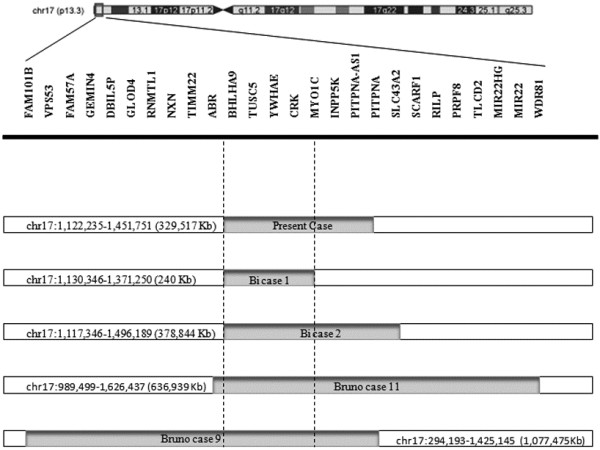
** Schematic representation of five 17p13.3 duplications in relation to gene content.** Enlargement of band p13.3 of chromosome 17. For each individual, the solid lines (grey shading) below the map represent duplicated regions and nucleotide positions are indicated according to UCSC Genome Browser Build 36.1 2009 (nucleotide positions of Bi cases 1 and 2 and Bruno cases 9 and 11 were converted from Hg18; nucleotide position of Bi case 1 was deduced (not indicated in the report). The dotted lines restrict the minimal critical region of overlap for 17p13.3 microduplications in the five cases.

Our patient and his mother have an interstitial microduplication of about 329.5 Kbs containing only seven genes. Many other patients with microduplications of varying size have been reported with different breakpoints and gene content [5,7,9]. Bruno et al. [5] reported on two cases (cases 9 and 11) in which duplicated regions overlapped the one reported here, containing *CRK*, *YWHAE,* and *BHLA9* genes. Only the duplication described by Bi et al., [9] (subject 2) is superimposable on that described here, while the duplicated region of subject 1 spans 240 Kb and contains only four genes: *TUSC5*, *YWHAE*, *CRK*, and *MYO1C* (Figure 4). Among the genes comprised in the duplicated region, particular interest has been paid to *YWHAE*. *YWHAE* is a gene encoding 14-3-3epsilon, which is highly conserved across species, from bacteria to humans, and binds to phosphoserine/phosphothreonine motifs in a sequence-specific manner. The individuals with duplications including YWHAE are characterized by a milder neurocognitive and pervasive developmental disorder phenotype, and share some minor craniofacial abnormalities [9]. The main phenotypic features of the patients include autistic manifestations, behavioral symptoms, developmental delay, varying degree of mental retardation, speech delay, several common facial features, and subtle hand/foot malformations. Three families were reported [10] with split-hand/foot malformation and long bone deficiency (SHFLD); the same authors suggested that a locus responsible for this condition is located within a duplicated segment on 17p13.3 band containing *ABR* and *BHLHA9* genes. In another study, seventeen patients with 17p13.3 duplication, among 56 families with SHFLD syndrome, showed a minimal critical region encompassing the *BHLHA9* gene [11]. Therefore, the authors concluded that the 17p duplication could be considered as a susceptibility locus for SHFLD, which is necessary but not sufficient for the development of these malformations. The high degree of non-penetrance could be dependent on other modifiers not identified yet. 

Bruno et al. [5] suggested that there are two classes of co-locating microduplications on 17p13.3. Class I duplications involve *YWHAE*, but not *PAFAH1B1* and the patients show autistic manifestation and behavioral problems, speech and motor delay, mild dysmorphic facial features, subtle hand and foot anomalies, and tendency to overgrowth. Class II microduplications always involve *PAFAH1B1* and may extend to *CRK* and *YWHAE* and have been associated to hypotonia, mild developmental, and psychomotor delay. Some dysmorphic features as prominent forehead and pointed chin, sometimes associated with microcephaly and growth restriction, are the most common characteristics [5]. Our patient is tall with mild facial anomalies like upslanting eyes, squared nasal tip, normal chin, large and low-set ears, short hands with low-set thumbs, while the feet had laterally set fifth toes. Moderate mental retardation was associated with a pervasive developmental disorder not otherwise specified (PDD-NOS) with social interactions and communication impairment, motor stereotypes, perseveration behaviours, and attention deficit. Brain MRI identified the presence of non-progressive neuroradiological features characterized by posterior corpus callosum hypoplasia and mild cerebellar hypoplasia. According to the identified duplication involving *YWHAE*, but not *PAFAH1B1*, our patient could fit as having a class I duplication. Furthermore, his clinical phenotype seems to overlap the features previously identified in such patients, as showed in Table 1. 

**Table 1:** Phenotypic features of patient with 17p13.3. class I microduplication

	**Index case**	**Bi et al., subject 1**	**Bi et al., subject 2**	**Bruno et al., case 9**	**Bruno et al., case 11**
Age	8 years	6 years	8 years	2 years	14 years
Gender	M	M	F	M	M
Gestational age (weeks)	37	NA	At term	At term	At term
Birth weight (g)	3350	3900	4592	3400	3487
Birth length (cm)	NA	NA	57	51	N
Birth head circumference (cm)	NA	NA	NA	NA	50^th^ centile
Postnatal growth retardation	-	-	-	-	-
Overgrowth	+ (75^th^ centile)	+ (90^th^ centile)	+3 SD	-	+ (90-97^th^ centile)
Feeding difficulties	-	-	-	-	+
Muscle hypotonia	+	+	+	-	+
Delay in motor function	+	+	+	Delay in fine motor skills	+
Cognitive development	Global delay	Mild to moderate global delay	Global delay	Normal at 2 years	Mild delay
Speech delay	++	++	++	+	+
Neurobehavioural symptoms	PDD-NOS	Behavior problems, aggressive tendencies	Behavior problems particularly with food	Autism, hyperactivity	Autism trait, facial tic
Repeated infection	-	NA	NA	-	-
Facial features:					
Face	Triangular			Triangular	N
Forehead	Broad		Broad	Broad	N
Eyes	Thick eyebrows, upslanting palpebral fissures	Thick eyebrows, synophrys	Upslanting palpebral fissures, synophrys	N	Broad, sparse eyebrows
Nose	Squared upturned tip of the nose	Squared, overhanging columella	Squared	N	Squared, upturned tip
Ears	Large	Large	Large	Prominent	Large, fleshy
Mouth	Thin	Thin upper lip	Thin upper lip	Prominent cupid bow	Prominent cupid bow
Mandible	Pointed chin	NA	Prominent chin	Pointed chin	Pointed chin
Hands/feet anomalies	Short and stubby with low-set thumbs	Large hands, small distal phalanges	Large hands	Bilateral groove between toe one and two	Hallux valgus, sandal gap, abnormal toe nails
Associated malformations	-	-	-	-	Genu valgum
MRI	Corpus callosum hypoplasia and mild cerebellar hypoplasia	NA	Thin corpus callosum	NP	NP

*NA* not available, *N* normal, *PDD-NOS* pervasive developmental disorder not otherwise specified, *NP* not performed.

However, in the patient’s family history we have to consider that his mother presented antisocial behaviour, bipolar disorder, and alcoholism, but unfortunately she was not available for clinical evaluation or MRI tests. Furthermore, his father was drug addicted and his grandmother was affected by depression. Several environmental problems, intertwining with genetic factors, affect this family. Although genetic and environmental influences may work independently, research is beginning to acknowledge that these factors work in concert to influence the behavioral phenotype, as depression and anxiety [12]. We should therefore consider the 17p13.3 duplication in terms of genetic contribution to a phenotype that, especially in his mother, seems to be due to different components.

## Conclusions

In conclusion, our report contributes to a better definition of the minimal critical region for class 1 microduplications including *BHLHA9*, *YWHAE*, and *CRK*. Additional patients will be necessary to further substantiate the significance of 17p13.3 microduplications and to establish a better genotype-phenotype correlation.

## Consent

Written informed consent was obtained from the patient for publication of this case report and any accompanying images. A copy of the written consent is available for review by the Editor-in-Chief of this journal.

## Competing interests

The authors declare that they have no competing interests related to this manuscript.

## Authors’ contributions

All authors reviewed the manuscript critically for its content, revised and edited it, and approved the final version.

## Pre-publication history

The pre-publication history for this paper can be accessed here:

http://www.biomedcentral.com/1471-2350/13/93/prepub
